# Treatment of Gonorrhœa

**Published:** 1873-04

**Authors:** 


### TREATMENT OF GONORRHŒA.

Mr. J. L. Milton, of London, in his late work on gonorrhœa, divides the means of treatment into three, viz.: internal remedies, external applications, and direct applications. Under the first division he discusses the use of copaiba, stating that though it has proved of service in a great many cases, yet it fails in a certain number in every form, and that severe symptoms have been produced from its use, "in doses which very good surgeons have not hesitated to recommend." He gives a table of cases treated with copaiba by him, with very unfavorable results.

He is favorably inclined toward the use of sandal wood oil combined with injections, but states that it is extremely difficult to get it pure. The other remedies contained in this section he condemns. He makes a tirade against antiphlogistic means, such as blood-letting, which seems to us to be at least twenty years behind date, as we know of no practice requiring to be thus declaimed against. A powerful purgative given at the outset of an attack, he believes, may be a material aid in cutting short the disease in mild cases; and aperients given along with injections, he thinks, may do good in many cases. It may here be remarked that this treatment, with the addition of the parts being supported, was prescribed at the out-door dispensary of the Glasgow Royal Infirmary by one of our professors, and it met with complete success in the greater number of cases. He thinks that diuretics may be of use. Mercurials he condemns, stating that they are still believed in in Germany, Belgium and Italy.

The only thing recommended by him under the head of external applications is hot water, so hot as to cause the penis to turn red. This, he states, gives great relief.

With regard to direct applications, he characterizes injections as the right arm of the service, and disputes the statement made by many that injections induce orchitis or stricture, at the same time admitting that they may hasten the appearance of the swelling in orchitis, though they cannot be considered the predisposing cause.

With regard to the substances to be injected, he gives a list of forty-six, which have been proposed within the last few years, many of them wholly injurious. A number of these he discusses at length, but prefers nit. argent. and chloride of zinc. He then gives his own treatment, which he divides into abortive and ordinary.

The abortive treatment may be used in cases adapted for it, which he describes. It consists of nit. argent. gr. v. to gr. x. to the ounce, introduced well into the urethra by a syringe with a long nozzle. The exhibition of gr. iv. calomel, followed by seidlitz powders until several loose motions are produced, and after each motion patient to inject urethra with nit. argent. gr. iii. to v. to the ℥j. He states that few cases are found suitable for the abortive treatment. His ordinary treatment consists in administering acetate of potash in combination with spirits of nitrous ether, along with purgatives and aperients, and injections of nit. argent., commencing at gr. ½ to gr. j. to ℥j., gradually increased to gr. x. to the ℥j., to be used once daily until the disease is almost gone, then every second day, and this to be continued for at least eight days after the last drop of discharge has shown itself. Along with this, the patient may use an injection of the sulphate and chlor. of zinc, 2 grs. of the former to ¼ gr. of the latter to the ℥j. And he lays down as a golden rule in using injections, "a slight feeling of heat for quarter of an hour or twenty minutes after is all that's required." If more than this, the injection is too strong; if less, it is too weak.

In women, he recommends the same internal treatment along with injections of sulph. of zinc, and, after a time, of oak bark.—*Medical and Surgical Reporter.*

